# Effects of Platelet TiB_2_ on the Formation and Mechanical Properties of (Zr,Ti)B_2_ Ceramics Prepared by Spark Plasma Sintering

**DOI:** 10.3390/ma19050946

**Published:** 2026-02-28

**Authors:** Shaolei Song, Peiqi Jiang, Yuanyuan Liu, Lei Lei, Yan Li

**Affiliations:** 1 School of Civil Engineering, Xuzhou University of Technology, Xuzhou 221018, China; 18018218098@163.com (P.J.); e_i27094@163.com (L.L.); 2 Research Center of Nano Science and Technology, Shanghai University, Shanghai 200444, China; 3 School of Materials and Chemical Engineering, Xuzhou University of Technology, Xuzhou 221018, China; lyy8email@163.com

**Keywords:** zirconium diboride, titanium diboride, platelet, spark plasma sintering, solid solution, mechanical properties

## Abstract

(Zr,Ti)B_2_ ceramics with enhanced hardness and fracture toughness were prepared by spark plasma sintering using platelet TiB_2_ and irregular ZrB_2_ as starting powders. The effects of sintering temperature (1700–1900 °C) and platelet TiB_2_ content (0–30 wt.%) on the sinterability, phase composition, microstructure, and mechanical properties of the (Zr,Ti)B_2_ ceramics were investigated. With increasing sintering temperature, the relative density of the solid solution increased from 89.9 ± 0.5% at 1700 °C to 97.7 ± 0.4% at 1800 °C, followed by no significant change upon further temperature elevation; however, the relative density showed an initial increase and subsequent decrease with increasing TiB_2_ content. Under optimized parameters (1800 °C, 3 min, 50 MPa, with a TiB_2_ content of 30 wt.%), (Zr,Ti)B_2_ ceramics achieve a maximum hardness of 24.9 ± 1.0 GPa, a fracture toughness of 5.0 ± 0.3 MPa·m^1/2^, and a relative density of 96.5 ± 0.5%. The high content of platelet TiB_2_ refined the (Zr,Ti)B_2_ grain size, reducing the D50 by 25.8% to 1.70 μm compared to the 20 wt.% content. This study provides a novel perspective for the design and preparation of high-performance ceramics.

## 1. Introduction

Ultra-high-temperature ceramics (UHTCs), as key candidate materials for application in extreme environments, have received widespread attention in recent years in fields such as aerospace and advanced manufacturing [1,2,3]. Among them, zirconium diboride (ZrB_2_) is considered one of the most promising ultra-high-temperature structural materials due to its excellent comprehensive properties, such as an extremely high melting point (~3245 °C) [4], good high-temperature strength [5], high thermal and electrical conductivity [6], and moderate density [7,8].

However, similar to other transition metal diborides, ZrB_2_ exhibits poor sinterability due to its inherent strong covalent bonds, oxygen impurities on particle surfaces, and relatively low self-diffusion coefficient [2]. The densification of ZrB_2_ often requires high temperatures and external pressure [9,10]. To overcome this challenge, researchers have explored various strategies, including the addition of sintering aids and second phases (e.g., SiC [11], ZrSi_2_ [12], B_4_C [13], TaB_2_ [14], TiB_2_ [15]). Among these, titanium diboride (TiB_2_) has emerged as a promising candidate due to its ultra-high-temperature ceramic nature and hexagonal AlB_2_-type crystal structure, which is identical to that of ZrB_2_. This structural similarity enables TiB_2_ to form a continuous solid solution system (Zr,Ti)B_2_ with ZrB_2_, thereby significantly improving the sintering performance of the ZrB_2_ matrix through solid solution strengthening [16]. This approach not only promotes densification but also optimizes mechanical and high-temperature properties by regulating the phase composition and microstructure [16]. S. Chakraborty et al. [17] investigated the variation in mechanical properties of ZrB_2_-TiB_2_-based ceramics with varying TiB_2_ content at 2200 °C using hot pressing. Their results revealed that the ZrB_2_-TiB_2_ ceramic with 30 wt.% TiB_2_ exhibited the highest hardness of 22.34 GPa and a fracture toughness of 3.01 MPa∙m^1/2^, along with good friction and wear performance. Similarly, Eric W. Neuman et al. [18] prepared ZrB_2_-based solid solutions with a relative density of 96% under hot pressing at 2100 °C by adding TiB_2_ (up to 50 vol%). They found that the addition of TiB_2_ significantly refined the grain structure and reduced thermal conductivity. Yuan et al. [19] further demonstrated the grain-refining effect of optimal TiB_2_ incorporation, demonstrating that the solid solution obtained after SPS sintering at 2000 °C showed significantly improved densification and Vickers hardness. Currently, research on TiB_2_-reinforced ZrB_2_ solid solution ceramics mainly uses particulate or equiaxed TiB_2_ as an additive. Previous studies have generally shown that the use of appropriate amounts of TiB_2_ can effectively refine the grain structure, lower the sintering temperature, and improve mechanical properties. However, systematic studies on the influence of anisotropic structure particles (such as two-dimensional platelet TiB_2_) on the properties of ZrB_2_ ceramics are still insufficient. Compared to granular phases, platelet second phases can induce various toughening mechanisms (such as crack deflection, bridging, and pull-out effects) in the matrix, theoretically providing better potential for coordinating material strength and toughness [20,21,22,23]. However, anisotropic structures in sintered samples may form uneven pore structures in the later stage of sintering due to excessive doping, which in turn affects the densification effect and ultimately affects the comprehensive mechanical properties of the solid solution [23]. Therefore, it is necessary to strictly control the platelet particle content to achieve performance optimization.

In this study, solid solution ceramics were prepared via spark plasma sintering (SPS) under vacuum conditions using ZrB_2_-TiB_2_ systems with varying platelet TiB_2_ content (0, 10, 20, and 30 wt.% relative to ZrB_2_). The influence of sintering temperature and TiB_2_ content on the sinterability, phase structure evolution, microstructure and mechanical properties of the obtained (Zr,Ti)B_2_ ceramics were investigated. Under the optimized process conditions of 1800 °C, insulation for 3 min, and external pressure of 50 MPa, the 30 wt.% TiB_2_ sample achieved the highest hardness (24.9 ± 1.0 GPa) and fracture toughness (5.0 ± 0.3 MPa∙m^1/2^). The excellent mechanical properties were attributed to the synergistic effect of microstructure control and multi-fracture behavior.

## 2. Materials and Methods

Commercially purchased ZrB_2_ micro-powder (purity ≥ 99.5%, particle size < 5 μm, Shanghai Xiangtian Nanomaterial Co., Ltd., Shanghai, China) and self-made platelet TiB_2_ (average thickness ~0.2 μm, lateral dimensions ~8 μm) were used as raw materials. Here, the self-made platelet TiB_2_ was obtained based on previous work [24], and the phase structure and microstructure can be found in Figure S1.

First, TiB_2_ and ZrB_2_ powders were weighed and placed in batches (with TiB_2_ mass fractions relative to ZrB_2_ of 0 wt.%, 10 wt.%, 20 wt.%, and 30 wt.%) into an agate mortar. Manual grinding was used to obtain a mixed powder, and the grinding process was conducted in an ethanol medium for 0.5 h to ensure uniform mixing. Subsequently, the mixtures were dried in a vacuum oven at 80 °C. After lining a Φ15 mm graphite mold with graphite paper, the mixed powders were uniformly poured into the mold and placed at the center of the SPS furnace plunger. The sintering process was carried out in a chamber with a vacuum level below 1 × 10^−3^ Pa following a three-stage program: (1) heating to 900 °C (with a rate of 50–100 °C/min) while maintaining a constant pressure of 1 MPa; (2) heating at a rate of 100 °C/min to 1100 °C, during which the pressure increased linearly from 2 MPa to 50 MPa; (3) rapidly heating at a rate of 200 °C/min to the target sintering temperature (1700 °C, 1800 °C, or 1900 °C), maintaining a constant pressure of 50 MPa, holding for 3 min, then removing the pressure and allowing the samples to cool rapidly with the furnace. To eliminate residual stresses, the sintered samples were buried in graphite powder and heat-treated at 1400 °C for 2 h under argon protection. Finally, the sample surfaces were polished using a grinding and polishing machine with progressively refined diamond grinding slurries.

The crystalline phases of the raw materials and sintered samples were analyzed using an X-ray diffractometer (XRD, Rigaku SmartLab, Tokyo, Japan) with Cu Kα radiation (λ = 1.5406 Å), operating at 40 kV and 40 mA. The scanning range was 10° to 80°, with a scan speed of 5°/min. The lattice parameters *a*_0_ and *c*_0_ of the sintered samples (with a crystal structure of the hexagonal crystal system) were determined using Equation (1). Subsequently, the Nelson–Riley extrapolation method [25] was employed to obtain *F*(*θ*) for different crystal planes under various diffraction angles. The parameters *a*_0_, *c*_0_, and *F*(*θ*) were plotted and linearly fitted to derive the corrected lattice parameters *a* and *c* of the sintered products.

(1) $$\frac{1}{d^{2}}=\frac{4(h^{2}+hk+k^{2})}{{3a}^{2}}+\frac{l^{2}}{c^{2}}$$

(2) $$F(\theta )=0.5(\frac{\cos^{2}\theta }{\sin\theta }+\frac{\cos^{2}\theta }{\theta })$$

Here, *d* represents the interplanar spacing; *h*, *k*, and *l* are the Miller indices; *a* and *c* are the lattice parameters; and *θ* is the diffraction angle, expressed in radians.

The surface morphology of the samples was observed using a field-emission scanning electron microscope (SEM, JEOL JSM-7800F, Tokyo, Japan). Energy-dispersive spectroscopy (EDS, Oxford X-Max, Oxford Instruments, Abingdon, UK) was used for elemental analysis. The test conditions were an accelerating voltage of 15 kV and beam current of 10 mA. The samples were sputter-coated with gold prior to testing. The backscattered electron (BSE) mode was utilized to enhance grain boundary contrast.

The relative density (D) of the sintered samples was measured using the Archimedean immersion method [26]. The calculations were performed according to Equations (3) and (4):

(3) $$\rho_{s}\;=\;\frac{m_{1}}{m_{3}-m_{2}}\rho_{\mathrm{water}}$$

(4) $$D=\frac{\rho_{s}}{\rho_{0}}\times 100\%$$

Here, *ρ_s_* is the sample volume density, *ρ_water_* is the density of deionized water, and *ρ*_0_ is the theoretical density of the sample; *m*_1_ is the mass of the dry sample in air, *m*_2_ is the mass of the sample when saturated in deionized water, and *m*_3_ is the mass of the sample in air after saturation in deionized water.

The Vickers hardness (HV) was measured using an HVS-10Z hardness tester (Laizhou Huayin, Laizhou, China), with a load of 98 N and a holding time of 15 s. Five parallel samples were used for each parameter, five points were tested on parallel samples, and the average value was calculated. The Anstis equation was used to calculate the indentation fracture toughness (*K_IC_*, MPa·m^1/2^) of the ceramics [27,28,29]:

(5) $$K_{\mathrm{IC}}\;=\;0.0889{\left(\frac{H_{V}\times P}{4l}\right)}^{1/2}$$

Here, *H_V_* is the Vickers hardness (GPa), *P* is the applied load force (in N), and *l* is the crack propagation length (m).

## 3. Results and Discussion

### 3.1. The Influence of Sintering Temperature on (Zr,Ti)B_2_ Ceramics

**Figure 1:**
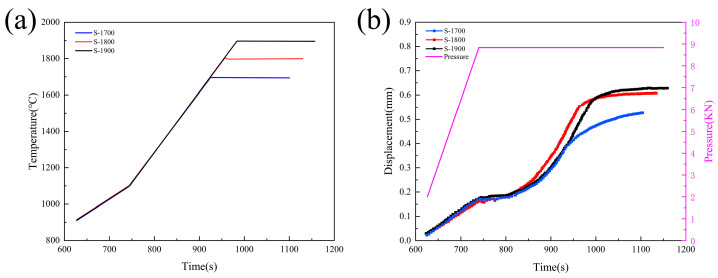
SPS sintering curves of the samples. (**a**) Temperature–time curve; (**b**) curves of pressure and compression displacement vs. time.

To determine the appropriate sintering temperature for achieving densification, Figure 1 presents the temperature–time, displacement–time, and pressure–time curves of the samples sintered at three different temperatures. As shown in Figure 1a,b, during the low-temperature stage (900–1100 °C), all three samples were heated at a reduced rate, with pressure gradually increasing and displacement changes exhibiting an approximately linear trend. At a constant pressure of 50 MPa (temperature between 1100 and 1400 °C), no significant displacement changes were observed, indicating that densification has not yet begun, which is consistent with previously reported results [17]. When the temperature exceeded 1400 °C, distinct densification behaviors emerged among samples S-1700, S-1800, and S-1900. A sharp increase in the slope of the displacement curve was recorded, marking the onset of rapid sintering and a substantial rise in densification kinetics. Further results revealed that the displacement of sample S-1700 continued to increase monotonically throughout the holding period, suggesting incomplete densification due to insufficient dwell time. In contrast, both S-1800 and S-1900 exhibited clear displacement plateaus during the isothermal phase, indicative of near-complete densification. However, the differing times at which these plateaus were reached imply variations in densification rates between the two samples. Quantitative evaluation based on the displacement change rate (Figure S2) demonstrated that S-1800 exhibited a higher rate during the intermediate sintering stage than S-1900, reflecting faster densification kinetics, whereas S-1900 showed a relatively low rate prior to attaining the densification plateau. Overall, the densification process evolved from a slow heating regime to a rapid densification stage, with notable differences in both densification kinetics and final densification states across the samples.

**Figure 2:**
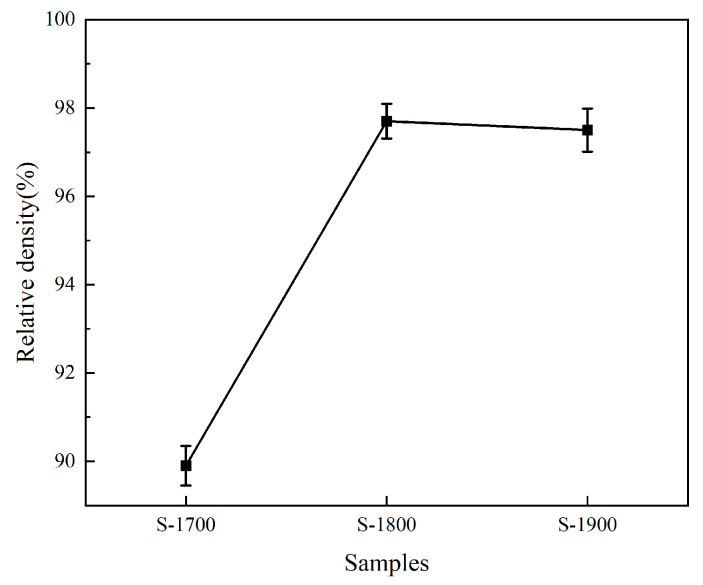
Relative density of samples under different sintering temperatures.

Figure 2 illustrates the influence of sintering temperature on the densification behavior of the (Zr,Ti)B_2_ solid solution. Experimental results indicate that the S-1700 sample sintered at 1700 °C achieved a relative density of only 89.9 ± 0.5%, consistent with the observed displacement patterns. This phenomenon is attributed to an insufficient sintering temperature, which results in a low particle mass transport coefficient, hindering effective pore closure and grain boundary migration within the limited sintering duration, thereby impeding the densification process [30]. In contrast, the S-1800 sample achieved relative densities of 97.7 ± 0.4%, indicating that this temperature range satisfies the complete densification requirements of (Zr,Ti)B_2_. During the SPS sintering process, the high specific surface area of platelet TiB_2_ significantly promotes interfacial reaction activity. The B atoms on the surface of ZrB_2_ particles undergo rapid atomic exchange with the Ti atoms in the TiB_2_ lattice, forming (Zr,Ti)B_2_ nuclear embryos through solid-state reaction diffusion. As the temperature increases, the SPS pulse current triggers a local Joule heating effect, accelerating the atomic migration rate at the grain boundary and causing the relative density to jump from 89.9 ± 0.5% to 97.7 ± 0.4%. Notably, no significant difference in density (97.5 ± 0.5%) was observed when the sintering temperature increased from 1800 °C to 1900 °C, suggesting that the densification process reaches thermodynamic equilibrium at 1800 °C, with density tending to stabilize. Furthermore, further increasing the sintering temperature may induce abnormal grain growth, which could degrade the material’s mechanical properties [31].

**Figure 3:**
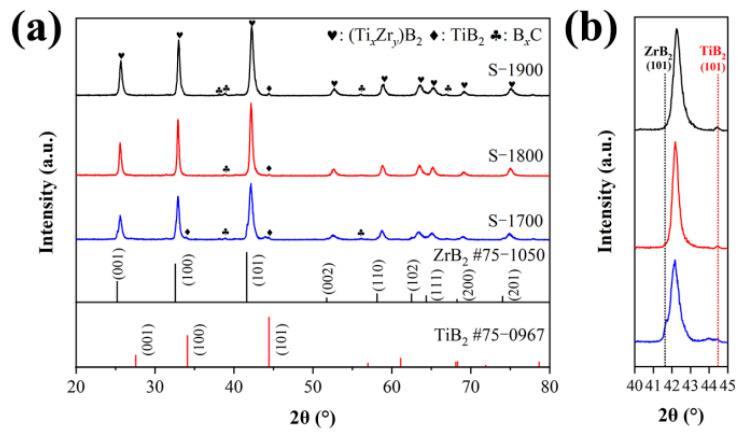
XRD patterns of samples at different sintering temperatures: (**a**) full patterns; (**b**) patterns from 40° to 45°.

Figure 3 presents the phase composition of the obtained samples sintered at different temperatures. As shown in Figure 3a, compared to the standard card, the characteristic diffraction peaks of the ZrB_2_ main phase in all samples exhibit a slight shift towards higher angles. This phenomenon is attributed to the formation of a solid solution structure between ZrB_2_ and TiB_2_ [18,32]. According to the Hume–Rothery solid solution rule, both ZrB_2_ and TiB_2_ belong to the hexagonal crystal system with the same space group (P6/mmm), and their similar crystal structures provide a structural basis for solid solution formation. Furthermore, the ionic radii of Zr and Ti are 0.72 Å and 0.605 Å, respectively, with a radius difference of approximately 15%, which falls within the critical range for continuous solid solution formation. Additionally, Zr and Ti have very similar electronegativities (Zr, 1.3, Ti, 1.2) and comparable chemical affinity for B, which reduces the interfacial energy barrier during the solid solution process and promotes lattice substitution. This solid solution behavior is consistent with the common second-phase regulation mechanism in ZrB_2_-based composite ceramics, such as in the ZrB_2_/B_4_C system [33], where second-phase particles can refine grain size through grain boundary pinning effects and also form composite structures through solid solution or reaction.

Figure 3b shows that the (101) crystal plane positions of the three samples shift towards higher angles with increasing temperature. Combined with the Nelson–Riley extrapolation method, the lattice parameters of the S-1800 sample were determined as *a* = 3.138 Å, *c* = 3.482 Å. Both values are slightly lower than those of ZrB_2_ (*a* = 3.17 Å, *c* = 3.53 Å) but higher than those of TiB_2_ (*a* = 3.028 Å, *c* = 3.2284 Å), indicating Zr atoms were partially replaced by Ti atoms and formed a (Zr,Ti)B_2_ solid solution. According to Bragg’s law (2d sin *θ* = *λ*), as Zr atoms are continuously replaced by Ti, the decrease in interplanar spacing d will cause diffraction peaks to shift towards higher angles. According to Vegard’s law [18], the corresponding solid solubility is calculated using the formula C = (x − a0)/(b0 − a0) × 100%, where a0 is the lattice parameter of pure ZrB_2_, b0 is the lattice parameter of pure TiB_2_, and x is the lattice parameter of the measured solid solution. The solid solubility of S-1800 is 22.5%. The further reduction in lattice parameters in the S-1900 sample indicates that the increase in temperature promotes a greater degree of substitution of Zr by Ti in the (Zr,Ti)B_2_ solid solution, with a solid solubility of 24.6%.

A comparative analysis of the phase changes in samples obtained at different temperatures reveals that the residual diffraction peaks of the ZrB_2_ (101) plane persist in the S-1700 sample but not in the S-1800 and S-1900 samples. This phenomenon indicates that the elevated temperature promotes the solid-state reaction between ZrB_2_ and TiB_2_. Simultaneously, a minor characteristic peak at 2θ = 44.48°, corresponding to the TiB_2_ (101) peak, suggests the presence of trace amounts of TiB_2_. These findings imply that under the SPS sintering process, rapid reactions can occur between micrometer-sized TiB_2_ and ZrB_2_ particles to form solid solutions. Additionally, the characteristic peaks observed in the BxC phases can be attributed to the high acceleration of atomic motion caused by pulse discharge and plasma. During spark plasma sintering, this phenomenon leads to the formation of BxC compounds from some B and C atoms [34]. Similar behavior was also observed in TiB_2_ bulk materials [35] (as shown in Figure S3).

**Figure 4:**
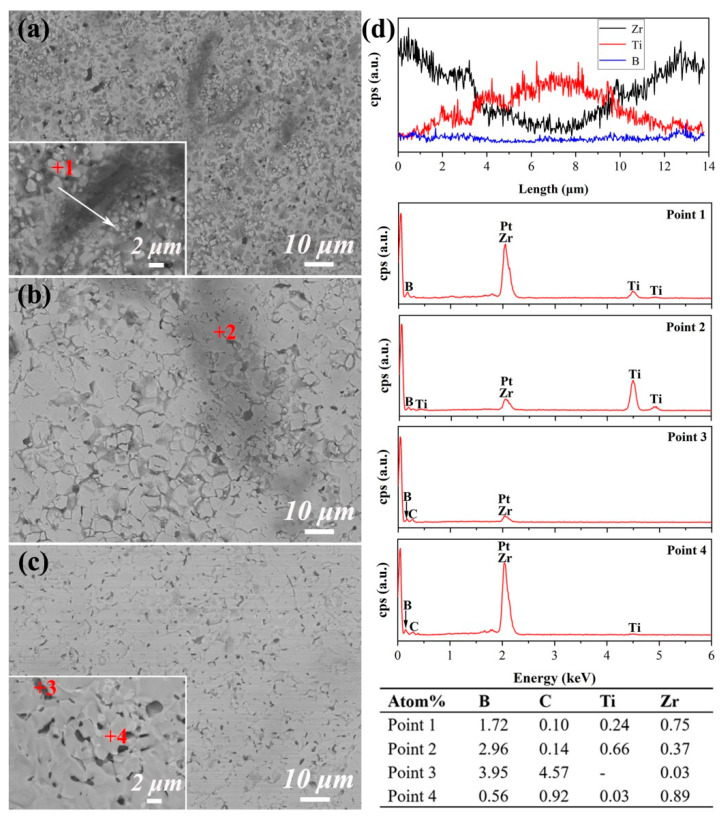
BSE images of the polished surface of the sample under different sintering temperatures: (**a**) S-1700; (**b**) S-1800; (**c**) S-1900; (**d**) EDS spectrum.

To investigate the impact of sintering temperature on the microstructure of (Zr,Ti)B_2_ ceramics, the polished surfaces of samples sintered at different temperatures were characterized using backscattered electron (BSE) imaging (Figure 4). As shown in Figure 4a, the microstructure of S-1700 comprises a dark-gray continuous phase with a dispersal of 1–2 μm. Some regions of the dark-gray phase exhibit anisotropic characteristics, and micropores (black areas) are also present. Based on the EDS line scan (white arrow) results, the dark-gray phase was identified as incompletely dissolved TiB_2_. The EDS results at point 1 indicate that the particle should be in the (Zr,Ti) B_2_ solid solution in the Zr-rich phase region. The line scan spectrum indicates Ti diffusion toward Zr-rich (Zr,Ti)B_2_ regions, suggesting that the solid solution reaction has initiated but is not yet complete. As the sintering temperature increases to 1800 °C (S-1800) and 1900 °C (S-1900), the pores and fine particle phases in the samples gradually disappear. The dark-gray phase becomes lighter. The scan of point 2, as shown in Figure 4b,d, confirms it as Ti-rich (Zr,Ti)B_2_, indicating that high temperatures promote densification and the solid solution reaction. This aligns with the principle that increased temperature reduces diffusion activation energy, thereby promoting atomic migration and pore elimination [36,37]. Additionally, trace carbide phases are detected in the samples (points 3 and 4 shown in Figure 4c,d), which are inferred to originate from carbon residue in the raw materials and diffusion contamination from the graphite paper of the sintering mold, consistent with the corresponding XRD results.

### 3.2. The Influence of TiB_2_ Content on (Zr,Ti)B_2_ Ceramics

**Figure 5:**
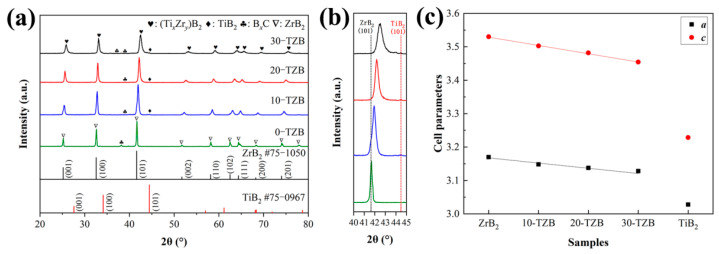
XRD patterns and cell parameters of the samples with different TiB_2_ content: (**a**) full patterns; (**b**) patterns from 40° to 45°; (**c**) cell parameters (*a*, *c*).

To investigate the influence of TiB_2_ content on the phase composition of the resulting products, XRD analysis was performed on samples with varying TiB_2_ content (Figure 5). As shown in Figure 5a, the main diffraction peaks of all TiB_2_-containing samples shift towards higher angles, further confirming the formation of (Zr,Ti)B_2_ ceramics. The enlarged spectrum of the strongest peak in Figure 5b clearly reveals that the (101) lattice plane position of the (Zr,Ti)B_2_ solid solution shifts towards higher angles with increasing TiB_2_ content, indicating an increase in solid solubility. The lattice parameters a and c, calculated using the Nelson–Riley extrapolation method (Figure 5c), demonstrate that as the TiB_2_ content increases, the a and c values of the solid solution linearly vary from the standard ZrB_2_ lattice parameters (*a* = 3.17 Å, *c* = 3.53 Å) towards those of TiB_2_ (a = 3.17 Å, *c* = 3.53 Å), consistent with Vegard’s law. This confirms that the Ti content in (Zr,Ti)B_2_ ceramics increases with increasing TiB_2_ content.

**Figure 6:**
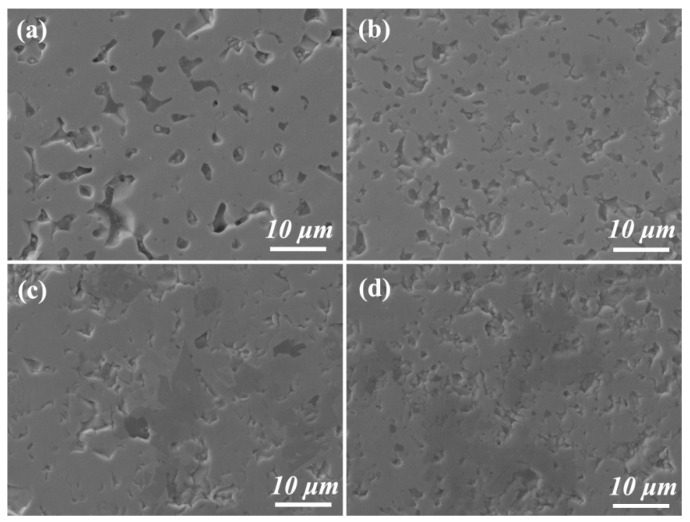
SEM images of the samples with different TiB_2_ content: (**a**) 0-TZB; (**b**) 10-TZB; (**c**) 20-TZB; (**d**) 30-TZB.

Figure 6 presents the SEM images of samples with varying TiB_2_ content. As the TiB_2_ content increases, the microstructure undergoes significant changes. In the 0-TZB sample (Figure 6a), numerous irregularly shaped and non-uniformly sized pores are observed, indicating a relatively low degree of densification within the material, which corroborates the sintering curve and the lower relative density. With the introduction of TiB_2_, the pore quantity is evidently reduced (Figure 6b–d). TiB_2_ particles can directly fill these pores to reduce porosity and increase material density. In addition, platelet TiB_2_ acts as heterogeneous nucleation sites, reducing the growth activation energy of ZrB_2_ grains, promoting grain boundary diffusion and grain rearrangement. This mechanism has been reported in ceramic composite systems where TiB_2_ serves as the second reinforcing phase. At the same time, the mutual dissolution of Ti^4+^ and Zr^4+^ in the hexagonal lattice forms (Zr,Ti)B_2_ ceramics, causing lattice distortion and enhancing atomic mobility, accelerating the densification process [38,39].

**Figure 7:**
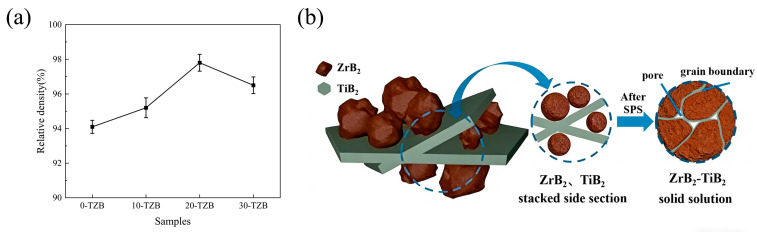
Variation in relative densities of samples with different TiB_2_ content: (**a**) relative densities; (**b**) densification process of interlaced platelet TiB_2_ with granular ZrB_2._

Figure 7 presents the variation in the relative density of the obtained samples with TiB_2_ content. As known from Figure 7a, the density of the samples exhibits a trend of first increasing and then decreasing with the increase in the content of platelet TiB_2_. The highest relative density (97.7 ± 0.4%) is achieved when the content of platelet TiB_2_ is 20 wt.%. However, further increasing the content of platelet TiB_2_ to 30 wt.% results in a decrease in the relative density of the sample to 96.5 ± 0.5% (30-TZB). This phenomenon may be attributed to the anisotropy of platelet TiB_2_ and its distribution structure within the sample. As shown in Figure 7b, some platelet TiB_2_ forms an interlaced structure. During the rapid sintering and densification process, an excess of such structures hinders the timely escape of pore gases, thereby impeding further densification of the sample. In addition, the cross-sectional particle size of the sample with a 30 wt.% addition was significantly smaller than that of the sample with a 20% addition, and the D50 of the particle size decreased from 2.29 microns to 1.70 microns (shown in Figure S4). This indirectly confirms the formation of more complex interwoven structures between platelet particles, hindering particle growth and leaving micropores, resulting in a decrease in density.

**Figure 8:**
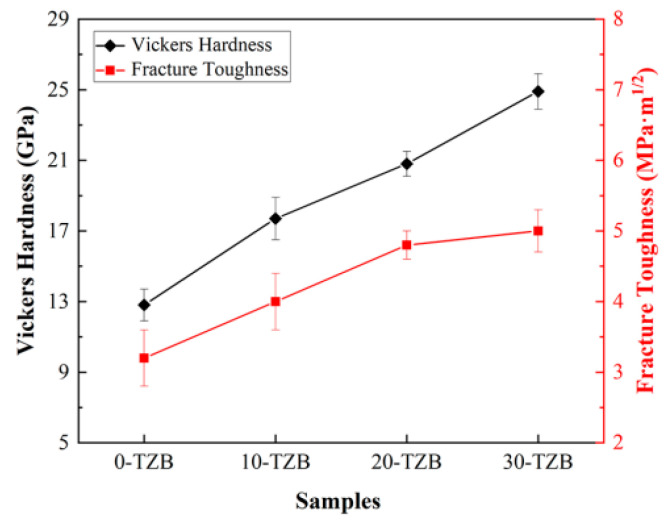
Vickers hardness and fracture toughness of samples with different TiB_2_ content.

Figure 8 presents the Vickers hardness and fracture toughness values of samples with different TiB_2_ content. It can be observed that as the TiB_2_ content increases from 0% to 30%, the Vickers hardness of the ceramic material exhibits a significant linear enhancement trend, rising from an initial value of 12.8 ± 0.9 GPa to 24.9 ± 1.0 GPa, an increase of nearly 100%. As a reinforcing phase, TiB_2_ has a very high hardness (25–35 GPa), which has a direct contribution to the enhancement of the hardness of ceramic composites [30]. According to the theory of solid solution strengthening, when forming a solid solution, Ti atoms replace Zr atoms to increase lattice distortion, effectively resisting dislocation motion and improving the strength and hardness of the matrix material [40].

Further analysis of the fracture toughness data reveals that the introduction of TiB_2_ significantly improves the fracture behavior of the ceramic. When the platelet TiB_2_ content reaches 30 wt.%, the fracture toughness value peaks at 5.0 ± 0.3 MPa·m^1/2^, representing a 56% improvement over the monolithic ZrB_2_ ceramic (0-TZB). Platelet TiB_2_ particles consume fracture energy during crack propagation through mechanisms such as bridging and crack deflection, thereby inhibiting rapid crack extension. Compared with literature-reported multiphase-toughened ZrB_2_ ceramics (Table 1), the platelet TiB_2_ toughening strategy employed in this study not only achieves a significant enhancement in fracture toughness but also maintains synchronous hardness improvement, breaking through the bottleneck in traditional multiphase materials where strength and toughness are difficult to optimize synergistically. The obtained materials have potential application prospects in extreme environmental engineering fields such as ultra-high-temperature structural materials, nuclear energy, and high-temperature electrode materials. The single-phase solid solution structure formed does not require the introduction of a second phase, avoiding interface weakening and thermal expansion mismatch problems while simplifying the preparation process and reducing costs. This provides a new avenue for the mechanical property regulation of ZrB_2_-based ultra-high-temperature ceramics.

### 3.3. Toughening Mechanism of Platelet TiB_2_

**Figure 9:**
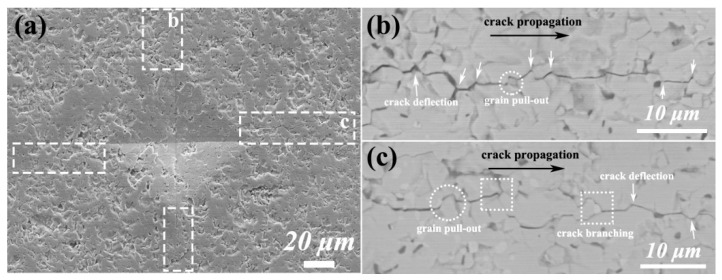
SEM images of the crack extension of sample 30-TZB: (**a**) SEM images of the cracks; (**b**,**c**) BSE images of the cracks in b and c areas from image (**a**).

To elucidate the toughening mechanism of TiB_2_ platelets in (Zr,Ti)B_2_ ceramics, Figure 9 presents the SEM images of crack propagation in the 30-TZB sample. As shown in Figure 9a, cracks generated via the indentation method exhibit a distinct “zigzag” propagation path (indicated by the white dashed box), indicating that the crack must overcome greater resistance [19]. Compared to cracks with a straight propagation path, the increased crack propagation path length significantly enhances the fracture resistance of the material [22]. To further reveal the underlying micro-fracture mechanism, Figure 9b,c displays backscattered electron images of the propagating crack. The crack undergoes significant deflection guided by grain boundaries (as indicated by the white arrows), forcing it to extend along these boundaries (i.e., intergranular fracture). This deflection process substantially increases the crack propagation path length, thereby consuming more fracture energy. The addition of platelet TiB_2_ powder acts as a grain boundary enhancer by forming a stable solid solution interface layer, which enhances intergranular cohesion. Furthermore, grain pull-out is clearly observable in the images (indicated by the white circular regions), indicating that the crack must overcome the intergranular bonding forces to “pull out” grains from the matrix during its passage through grain boundaries—a process requiring additional energy [44]. Grain pull-out was observed in all samples, which is consistent with findings reported in the literature [30,34]. Crack branching (indicated by the white square regions) decomposes a single main crack into multiple sub-cracks, significantly increasing the total newly created crack surface area and thus efficiently dissipating the energy at the crack tip.

**Figure 10:**
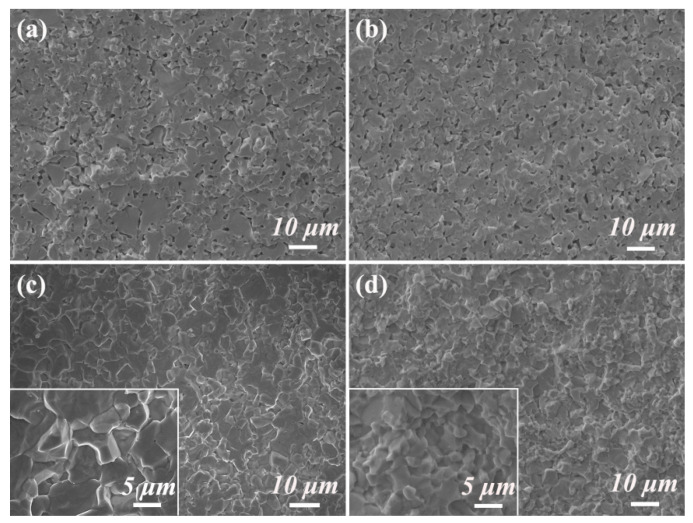
Fractional SEM images of samples with different additional TiB_2_ amounts: (**a**) 0% (0-TZB); (**b**) 10% (10-TZB); (**c**) 20% (20-TZB); (**d**) 30% (30-TZB).

Figure 10 illustrates the fracture cross sections of various samples. A comparative analysis of the fracture morphologies reveals that the 0-TZB and 10-TZB samples (Figure 10a,b) exhibit a higher number of pore defects than the 20-TZB and 30-TZB samples. This observation indicates that samples with lower TiB_2_ content exhibit relatively poorer density. Given that material density is directly correlated with fracture toughness, insufficient density generally results in reduced resistance to crack propagation. Consequently, this microstructural characteristic provides a rationale for the lower fracture toughness of the 0-TZB and 10-TZB samples.

Further observation of the cross-section images of the samples with high TiB_2_ content (20-TZB and 30-TZB) (Figure 10c,d) clearly identified grain boundaries and pits left after grain pull-out. This type of pit corresponds to the extraction of particles in Figure 9b,c, and its appearance requires additional energy consumption, which is also one of the important mechanisms for material toughening.

In addition, with the increase in TiB_2_ content, as shown in Figure S4, the particle size distribution of the four samples tends to be more concentrated, and the average particle size decreases. When the TiB_2_ content reaches 30 wt.% (30-TZB sample), compared with the 20-TZB sample, the average particle size decreases from 2.29 μm to 1.70 μm, and the particle size decreases by 25.8%. This phenomenon indicates that an increase in TiB_2_ content has the effect of refining grain size [45]. When solute Ti ions segregate at grain boundaries, this segregation reduces the interfacial energy of grain boundaries, thereby weakening the driving force and migration rate of grain boundary migration [17,19]. Therefore, an appropriate increase in TiB_2_ content can effectively suppress the growth of solid solution grains during the high-temperature sintering process. Grain refinement leads to an increase in the total grain boundary area, which enhances the strength of the (Zr,Ti)B_2_ [15,46]. Meanwhile, it consumes more energy by increasing the twisting (deflection and branching) of crack propagation paths, significantly improving the fracture toughness of the material [18,47].

## 4. Conclusions

This study successfully prepared (Zr,Ti)B_2_ ceramics using platelet TiB_2_ and irregular ZrB_2_ as raw materials through spark plasma sintering (SPS) technology. The influence of sintering temperature (1700–1900 °C) and TiB_2_ content (0–30 wt.%) on the densification behavior, microstructure, phase composition, and mechanical properties of solid solutions was studied. The main conclusions are as follows.

The sintering temperature has a significant promoting effect on the densification of solid solutions. When the sintering temperature was increased from 1700 °C to 1800 °C, the relative density of the material increased from 89.9 ± 0.5% to 97.7 ± 0.4%, an increase of 7.8%, indicating that the sintering process entered a rapid densification stage within this temperature range. Further increasing the temperature results in no significant change in relative density, indicating that almost complete densification has been achieved at 1800 °C.

As the TiB_2_ content increases from 0% to 30% by weight, the relative density of the solid solution shows a trend of first increasing and then decreasing. Although the density decreases after exceeding 20 wt.%, it still maintains a high density (96.5 ± 0.5%), indicating that the system still has good sintering properties. The hardness and fracture toughness continue to increase with the increase in TiB_2_ content. Under the optimized process conditions of 1800 °C, insulation for 3 min, and external pressure of 50 MPa, the 30 wt.% TiB_2_ sample achieved the highest hardness (24.9 ± 1.0 GPa) and fracture toughness (5.0 ± 0.3 MPa∙m^1/2^).

Adding an appropriate amount of platelet TiB_2_ particles can refine the grain size, and compared to a content of 20 wt.% TiB_2_, the D50 is reduced by 25.8% to 1.70 μm. The hardness and fracture toughness of the solid solution are improved through crack deflection, particle extraction, crack branching, and solid strengthening.

## Figures and Tables

**Table 1:** Comparison of related properties of several similar ceramic materials.

Sample	Sintering Process	Relative Density (%)	Vickers Hardness (GPa)	Fracture Toughness (MPa·m^1/2^)	Ref.
(Ti,Zr)B*_2_*	*HP*, 40 MPa, 2200 °C–2 h	97	22.3	3.0	[17]
(Ti,Zr)B*_2_*	*SPS*, 50 MPa, 2100 °C–15 min	97.5	20.6	4.7	[41]
(Ti*_x_*Zr_1−*x*_)B*_2_*–GNP	*SPS*, 50 MPa, 1800 °C–5 min	98.9	23.5	5.3	[42]
(Ti*_x_*Zr_1−*x*_)B*_2_* –(Zr*_x_*Ti_1−*x*_)N	*SPS*, 30 MPa, 2000 °C–8 min	99.2	28.0	8.5	[43]
20-TZB (20 wt.% TiB_2_)	*SPS*, 50 MPa, 1800 °C–3 min	97.8	20.6	4.8	This work
30-TZB (30 wt.% TiB_2_)		96.5	24.9	5.0	

## Data Availability

The original contributions presented in this study are included in the article/Supplementary Material. Further inquiries can be directed to the corresponding authors.

## Supplementary Materials

The following supporting information can be downloaded at: https://www.mdpi.com/article/10.3390/ma19050946/s1, Figure S1. XRD (a) and SEM images (b) of self-made platelet TiB_2_. Figure S2. Displacement change rate of the S-1800 and S-1900 samples during the SPS sintering process. Figure S3. SEM-EDS analysis of the gray phases formed in the TiB_2_ on a polished surface. Figure S4. Grain size of the solid solution with different amounts of TiB_2_: (a) 0%; (b) 10%; (c) 20%; (d) 30%.

## References

[1] Zhao R., Wang L.-M., Liu Q., Pang S.-Y., Liang B., Li J., Hu C.-L., Tang S.-F. (2025). Preparation, mechanical properties and long-term ablation behaviors of ultra-high temperature solid solution ceramic matrix composites. J. Alloys Compd..

[2] Singh K.P., Bajpai S., Balani K. (2025). Microstructure—Mechanical property correlation in multi-component dual-phase (Zr,Ti,Ta) boride-carbide ultra high temperature ceramic. Mater. Sci. Eng. A.

[3] Golla B.R., Mukhopadhyay A., Basu B., Thimmappa S.K. (2020). Review on Ultra High Temperature Boride Ceramics. Prog. Mater. Sci..

[4] Naughton-Duszova A., Bączek E., Podsiadło M. (2018). Selected properties of ZrB_2_ composites obtained by SPS method for parts of electro-erosion shaping machines. Mies. Nauk.-Tech..

[5] Fahrenholtz W.G., Hilmas G.E., Talmy I.G., Zaykoski J.A. (2007). Refractory Diborides of Zirconium and Hafnium. J. Am. Ceram. Soc..

[6] Hassan R., Balani K. (2021). Densification mechanism of spark plasma sintered ZrB_2_ and ZrB_2_-SiC ceramic composites. Mater. Charact..

[7] Zhang Y.-H., Lunghi A., Sanvito S. (2020). Pushing the limits of atomistic simulations towards ultra-high temperature: A machine-learning force field for ZrB_2_. Acta Mater..

[8] Wang R.-Z., Li W.-G. (2017). Determining fracture strength and critical flaw of the ZrB_2_–SiC composites on high temperature oxidation using theoretical method. Compos. Part B Eng..

[9] Gubernat A., Kornaus K., Zientara D., Zych Ł., Rutkowski P., Komarek S., Naughton-Duszova A., Liu Y.-S., Chlubny L., Pedzich Z. (2025). Modification of Thermo-Chemical Properties of Hot-Pressed ZrB_2_-HfB_2_ Composites by Incorporation of Carbides (SiC, B_4_C, and WC) or Silicides (MoSi_2_ and CrSi_2_) Additives. Materials.

[10] Bona E.D., Karacasulu L., Vakifahmetoglu C., Sglavo V.M., Biesuz M. (2024). Ultrafast high-temperature sintering (UHS) of WC and WC-containing ZrB_2_. J. Alloys Compd..

[11] Reimer T., Di Martino G.D., Sciti D., Zoli L., Galizia P., Vinci A., Lagos M.A., Azurmendi N. (2023). Experimental characterization of fatigue life of ZrB_2_-SiC based ultra high-temperature ceramic matrix composites. Int. J. Fatigue.

[12] Balbo A., Sciti D. (2008). Spark plasma sintering and hot pressing of ZrB_2_–MoSi_2_ ultra-high-temperature ceramics. Mater. Sci. Eng. A.

[13] Liu L.-L., Dou Z.-Y., Ran S.-L. (2025). Mechanical properties and microstructure of B_4_C–ZrB_2_–graphite composites fabricated by reactive spark plasma sintering. J. Alloys Compd..

[14] Demirskyi D., Vasylkiv O. (2017). Flexural strength behavior of a ZrB_2_–TaB_2_ composite consolidated by non-reactive spark plasma sintering at 2300 °C. Int. J. Refract. Met. Hard Mater..

[15] Yin J., Huang Z.-R., Liu X.-J., Yan Y.-J., Zhang H., Jiang D.-L. (2013). Mechanical properties and in-situ toughening mechanism of pressurelessly densified ZrB_2_–TiB_2_ ceramic composites. Mater. Sci. Eng. A.

[16] Li L.F., Zhang L., Tan C., Rong Y., Wang H.-B. (2025). Effects of TaC additives on the mechanical properties, microstructures, and oxidation behavior of ZrB_2_-SiC-TaC composites prepared by spark plasma sintering. Ceram. Int..

[17] Chakraborty S., Debnath D., Mallick A.R., Das P.K. (2014). Mechanical and thermal properties of hot pressed ZrB_2_ system with TiB_2_. Int. J. Refract. Met. Hard Mater..

[18] Neuman E.W., Thompson M., Fahrenholtz W.G., Hilmas G.E. (2021). Thermal properties of ZrB_2_-TiB_2_ solid solutions. J. Eur. Ceram. Soc..

[19] Yuan J.-H., Guo W.-M., Liu Q.-Y., Zhang Y., Wu L.-X., You Y., Sun S.-K., Bai M.-W., Lin H.-T. (2021). Influence of TiB_2_ and CrB_2_ on densification, microstructure, and mechanical properties of ZrB_2_ ceramics. Ceram. Int..

[20] Liu H.-T., Zou J., Ni D.-W., Wu W.-W., Kan Y.-M., Zhang G.-J. (2011). Textured and platelet-reinforced ZrB_2_-based ultra-high-temperature ceramics. Scr. Mater..

[21] Yue C.-G., Liu W.-W., Zhang L., Zhang T.-H., Chen Y. (2013). Fracture toughness and toughening mechanisms in a (ZrB_2_–SiC) composite reinforced with boron nitride nanotubes and boron nitride nanoplatelets. Scr. Mater..

[22] Liu J., Zou J., Qiu S.-H., Liu J.-J., Wang W.-M., Fu Z.-Y. (2025). Multifunctional and anisotropic Cf/ZrB_2_ based composites prepared via a combined injection and vacuum impregnation approach. J. Mater. Sci. Technol..

[23] Li X.-C., Zhang T., Chen C., Song S.-L., Shen S.-Y., He G.-Z., Li Z., Li R., Zhen Q., Bashir S. (2022). Preparation of TiB_2_–SiC composites toughened with interlocking microstructure by self-assembled TiB_2_ plates. Ceram. Int..

[24] Song S.-L., Zhang T., Xie C., Zhou J.-M., Li R., Zhen Q. (2020). Growth behavior of TiB_2_ hexagonal plates prepared via a molten-salt-mediated carbothermal reduction. J. Am. Ceram. Soc..

[25] Sengupta P., Sahoo S.S., Bhattacharjee A., Basu S., Manna I. (2021). Effect of TiC addition on structure and properties of spark plasma sintered ZrB_2_–SiC–TiC ultrahigh temperature ceramic composite. J. Alloys Compd..

[26] Shao G., Zhao X.-T., Wang H.-L., Chen J.-B., Zhang R., Fan B.-B., Lu H.-X., Xu H.-L., Chen D. (2016). ZrB_2_-ZrSi_2_-SiC composites prepared by reactive spark plasma sintering. Int. J. Refract. Met. Hard Mater..

[27] Shetty D.K., Wright I.G., Mincer P.N., Clauer A.H. (1985). Indentation fracture of WC-Co cermets. J. Mater. Sci..

[28] Shankar E., Balasivanandha Prabu S. (2017). Mi Influence of WC and cobalt additions on the microstructural and mechanical properties of TiCN-Cr_3_C_2_-nano-TiB_2_ cermets fabricated by spark plasma sintering. Int. J. Refract. Met. Hard Mater..

[29] Fabijanić T.A., Ćorić D., Musa M.Š., Sakoman M. (2017). Vickers Indentation Fracture Toughness of Near-Nano and Nanostructured WC-Co Cemented Carbides. Metals.

[30] Savari V., Balak Z., Shahedifar V. (2022). Combined and alone addition effect of nano carbon black and SiC on the densification and fracture toughness of SPS-sintered ZrB_2_. Diam. Relat. Mater..

[31] Yi H.-Q., Ren K.-Z., Chen H., Cheng X., Xie X.-L., Liang M.-T., Yin B.-B., Yang Y. (2024). Molten Aluminum-Induced Corrosion and Wear-Resistance Properties of ZrB_2_-Based Cermets Improved by Sintering-Temperature Manipulation. Materials.

[32] Liu X.-Z., Deng C.-M., Zhang X.-F., Duan X.-H., Zhao R.-M., Li S.-H. (2020). MTiB_2_–ZrB_2_–SiC composite ceramic coating with the formation of solid-phase (Ti_x_Zr_1-x_)B_2_ deposited by atmospheric plasma spraying as a barrier to molten cryolite-based salt. Ceram. Int..

[33] Yanmaz L., Sahin F.C. (2023). Investigation of the density and microstructure homogeneity of square-shaped B_4_C-ZrB_2_ composites produced by spark plasma sintering method. J. Eur. Ceram. Soc..

[34] Yin Z., Yuan J., Xu W., Liu K., Yan S. (2018). Graphene nanosheets toughened TiB_2_-based ceramic tool material by spark plasma sintering. Ceram. Int..

[35] Zhang T., Song S.-L., Xie C., He G.-Z., Xing B.-H., Li R., Zhen Q. (2019). Preparation of highly-dense TiB_2_ ceramic with excellent mechanical properties by spark plasma sintering using hexagonal TiB_2_ plates. Mater. Res. Express.

[36] Tripathi S., Bhadauria A., Tiwari A., Tiwari A.K. (2023). Effect of carbonaceous reinforcements on mechanical properties of ZrB_2_-SiC composites via nanoindentation study. Diam. Relat. Mater..

[37] Zdaniewski W.A. (1987). Solid Solubility Effect on Properties of Titanium Diboride. J. Am. Ceram. Soc..

[38] Cui K., Li Y.-K. (2016). Fabrication, mechanical properties and thermal shock resistance of laminated TiB_2_-based ceramic. Int. J. Refract. Met. Hard Mater..

[39] Zhang Z.-H., Yi H.-G., Liang M.-T., Xie L.-Y., Yin B.-B., Yang Y. (2024). Effect of Sintering Process on Microstructure and Properties of (Zr_0.2_Ta_0.2_Ti_0.2_Cr_0.2_Hf_0.2_)Si_2_ High-Entropy Silicide Ceramics. Coatings.

[40] Zhang J., Wang S., Li W. (2019). Consolidation and characterization of highly dense single phase Ta-Hf-C solid solution ceramics. J. Am. Ceram. Soc..

[41] Mondal S., Chakraborty S., Das S. (2018). Mechanical and tribological behavior of ZrB_2_-TiB_2_ system prepared by mechanical activation spark plasma sintering technique. J. Mater. Eng. Perform..

[42] Akarsu M.K., Akin I. (2020). Mechanical properties and oxidation behavior of spark plasma sintered (Zr,Ti)B_2_ ceramics with graphene nanoplatelets. Ceram. Int..

[43] Derakhshandeh M.R., Fazili A., Golenji R.B., Alipour F., Eshraghi M.J., Nikzad L. (2021). Fabrication of (Ti_x_Zr_1−x_)B_2_-(Zr_x_Ti_1−x_)N composites by reactive spark plasma sintering of ZrB_2_-TiN. J. Alloys Compd..

[44] Yuan J.-H., Liu Q.-Y., You Y., Zeng L.-Y., Bai M.-W., Blackburn L.R., Guo W.-M., Lin H.-T. (2021). Effect of ZrB_2_ powders on densification, microstructure, mechanical properties and thermal conductivity of ZrB_2_-SiC ceramics. Ceram. Int..

[45] Meng K., Huang C.-Z., Shi Z.-Y., Xu L.-H., Wang Z., Huang S.-Q., Qu M.-N., Xu Z.-K., Zhang D.-J., Guo B.-S. (2025). Effect of TiB_2_ on properties and microstructure of Si_3_N_4_-TiB_2_ ceramics sintered at lower temperature. Int. J. Appl. Ceram. Technol..

[46] Sulima I., Boczkal G. (2023). Processing and Properties of ZrB_2_-Copper Matrix Composites Produced by Ball Milling and Spark Plasma Sintering. Materials.

[47] Yao W.-K., Yan J.-B., Li X.-C., Chen P.-G., Zhu Y.-L., Zhu B.-Q. (2022). In Situ ZrB_2_ Formation in B_4_C Ceramics and Its Strengthening Mechanism on Mechanical Properties. Materials.

