# Supplementary material for: Effects of Platelet TiB2 on the Formation and Mechanical Properties of (Zr,Ti)B2 Ceramics Prepared by Spark Plasma Sintering
Source: Materials (Basel). 2026 Feb 28;19(5):946. doi: 10.3390/ma19050946 (PMC12986502; doi:10.3390/ma19050946)
Supplement: Supplementary file 1 [file materials-19-00946-s001.zip › materials-4160810-supplementary.pdf]

Supplemental Materials

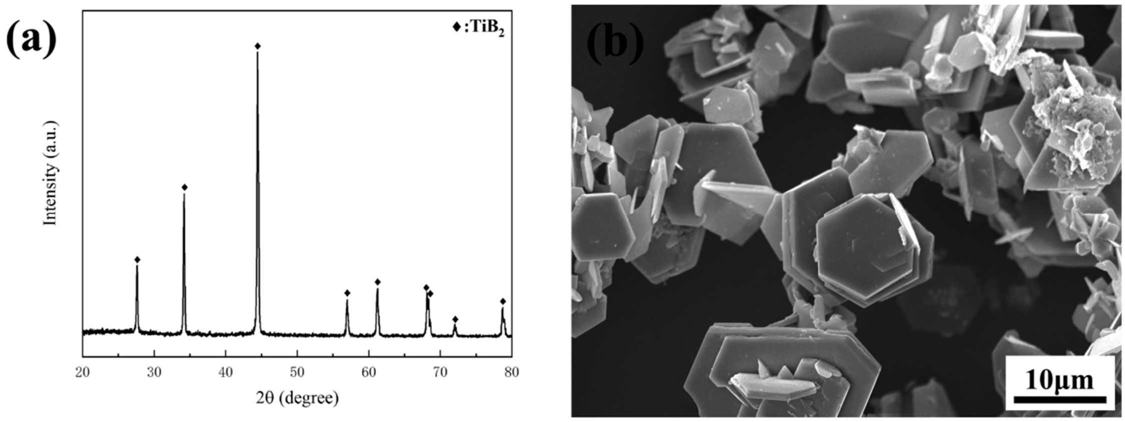

Figure S1. XRD (a) and SEM images (b) of self-made platelet TiB_2.

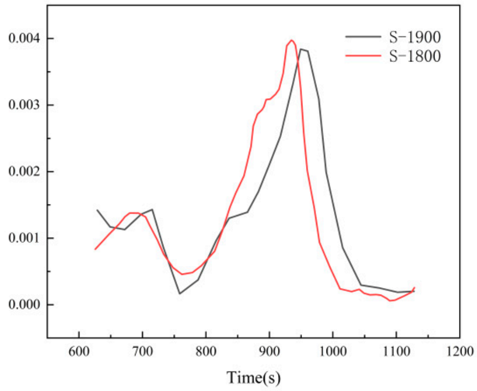

Figure S2 Displacement change rate of the S-1800 and S-1900 samples during the SPS sintering process.

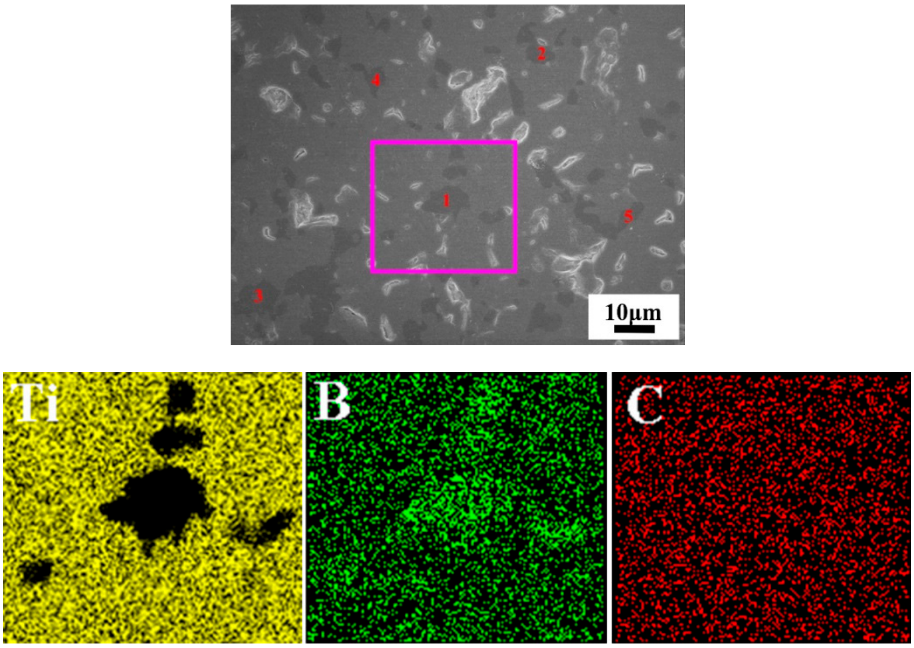

Figure S3. SEM-EDS analysis of the gray phases formed in the TiB_2 on a polished surface.

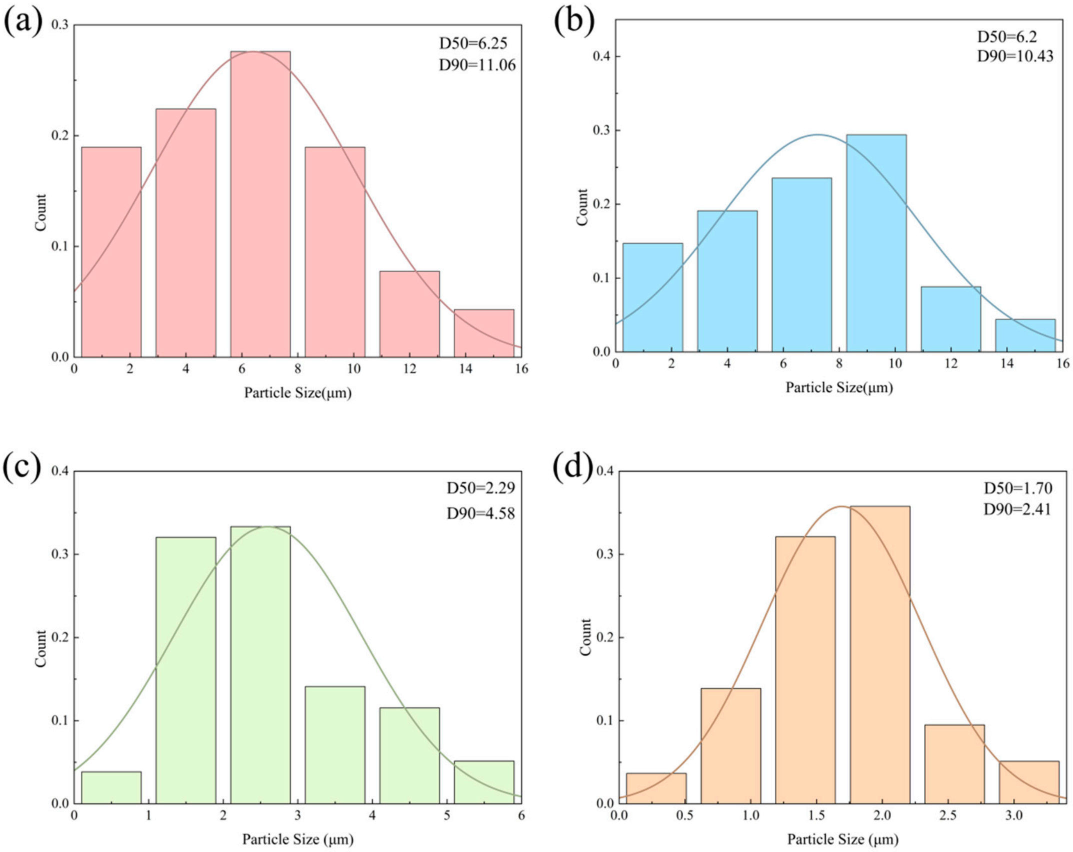

Figure S4. Grain size of the solid solution with different amounts of TiB_2: (a) 0%; (b) 10%; (c) 20%; (d) 30%.
